# Account of Diseases in an Eastern District of London, from the 20th of October to the 20th of November, 1807

**Published:** 1807-12

**Authors:** 


### *Account of Diseases in an Eastern District of London, from the 20th of October to the 20th of November,* 1807.

| | |
|---|---|
| **ACUTE DISEASES.** | |
| Typhus - - - | 2 |
| Ephemera - - | 3 |
| Cynanche tonsillaris - | 2 |
| Rubeola - - - | 6 |
| Quotidiana - - | 1 |
| Rheumatismus Acutus | 3 |
| **CHRONIC DISEASES.** | |
| Tussis - - - | 12 |
| Dyspnœa - - | 9 |
| Tussis cum Dyspnœa - | 10 |
| Asthma - - - | 1 |
| Hydrothorax - - | 2 |
| Hæmoptysis - - | 1 |
| Pleurodyne - - | 4 |
| Cephalalgia - - | 5 |
| Vertigo - - - | 2 |
| Paralysis - - - | 1 |
| Hysteria - - | 2 |
| Ascites - - - | 1 |
| Anasarca - - | 3 |
| Enterodynia - - | 4 |
| Diarrhœa - - - | 6 |
| Dysuria - - - | 2 |
| Rheumatismus Chronicus | 20 |
| **PUERPERAL DISEASES.** | |
| Convulsio - - | 1 |
| Ephemera - - | 3 |
| Menorrhagia Lochialis | 4 |
| **INFANTILE DISEASES.** | |
| Vermes - - - | 5 |
| Aphthæ - - - | 2 |
| Herpes - - - | 3 |
| Ophthalmia - - | 1 |

The measles have lately prevailed to a considerable degree, and in more than a few instances have proved fatal. Several children, in the same family, have fallen victims to the disease. In some cases, like other acute diseases, it has terminated fatally in a few days: a high degree of fever has preceded, or accompanied, the eruption; and the difficulty of breathing, and other pneumonic symptoms, have appeared in an aggravated form, and the patient has died of an inflammation of the lungs. But where these symptoms have been less violent, and the patient has past, without much alarm, through the different stages of the disorder, and even after all apprehension of danger in the mind of parents or friends has been dismissed, a continuance or recurrence of pneumonic symptoms has laid a foundation for phthisis pulmonalis.

The small-pox, another disease, the expectation of which formerly proved the occasion of constant anxiety and apprehension, has for some years, by the introduction of inoculation, been rendered much milder in its effects; and the hope of a total extermination of it has lately been entertained, in consequence of the introduction of Vaccination.

This disease, however, has lately appeared in a very malignant form; and the propagation of it has been comparatively extensive. If this be owing to a neglect of vaccivaccination, occasioned by the very few cases of small-pox, which have been supposed to occur after this preventive remedy has been employed, we cannot fail to lament the unwarrantable prejudice which has been entertained. If the few reports which have been circulated, have their foundation in a number of incontrovertible facts, yet how very small a proportion do they bear to the testimonies, which have been given to the utility of the practice on a widely extended scale, both at home and abroad.

